# Monitoring spawning migrations of potamodromous fish species via eDNA

**DOI:** 10.1038/s41598-019-51398-0

**Published:** 2019-10-28

**Authors:** Bettina Thalinger, Elisabeth Wolf, Michael Traugott, Josef Wanzenböck

**Affiliations:** 1 Department of Ecology, University of Innsbruck, Innsbruck, Austria; 2 Research Department for Limnology, University of Innsbruck, Mondsee, Austria; 3 Present Address: Centre for Biodiversity Genomics, University of Guelph, Guelph, Canada

**Keywords:** Animal migration, Freshwater ecology, Molecular ecology

## Abstract

Potamodromous fish are considered important indicators of habitat connectivity in freshwater ecosystems, but they are globally threatened by anthropogenic impacts. Hence, non-invasive techniques are necessary for monitoring during spawning migrations. The use of environmental DNA (eDNA) potentially facilitates these efforts, albeit quantitative examinations of spawning migrations remain so far mostly uncharted. Here, we investigated spawning migrations of Danube bleak*, Alburnus mento*, and Vimba bream, *Vimba vimba*, and found a strong correlation between daily visual fish counts and downstream eDNA signals obtained from filtered water samples analysed with digital PCR and end-point PCR coupled with capillary electrophoresis. By accounting for daily discharge fluctuations, it was possible to predict eDNA signal strength from the number of migrating fish: first, the whole spawning reach was taken into account. Second, the model was validated using eDNA signals and fish counts obtained from the upper half of the examined river stretch. Consequently, fish counts and their day-to-day changes could be described via an eDNA-based time series model for the whole migration period. Our findings highlight the capability of eDNA beyond delivering simple presence/absence data towards efficient and informative monitoring of highly dynamic aquatic processes such as spawning migrations of potamodromous fish species.

## Introduction

Generally, three main types of fish migration patterns are distinguished according to the type of water body inhabited: while diadromous and oceanodromous migrations commonly receive more attention due to the economic relevance of taxa such as salmon, tuna, and eel, the importance of potamodromous fish migrating solely within freshwaters has been frequently overlooked^1^. Nevertheless, these species can be key for the functioning of river ecosystems by influencing nutrient cycling and energy transfer between ecosystems^2^. Main drivers for potamodromous migrations are feeding, refuge-seeking (e.g. overwintering), and spawning^1^. The continuity of a river, associated with the possibility to reach suitable habitats, is crucial for maintaining sustainable populations of these mostly iteroparous species, characterised by multiple non-lethal spawning migrations during their lifetime^3,4^. In regions such as Europe, where 74% of river stretches are classified as “strongly affected” by channel fragmentation and water flow regulation^5^, tracing and quantifying the remaining spawning migrations of potamodromous fish species is highly efficient for monitoring aquatic ecosystem connectivity and the status of these often-threatened species.

As it is of particular importance to minimise disturbance at this sensitive stage of the life cycle^1^, numerous conventional methods such as electrofishing, mark-recapture, tags (incl. telemetry), trapping, netting via fish-counting fences or determining stable isotope ratios in fish tissues are not suitable due to their invasiveness^6^. Thus, visual observations from outside the river or by snorkelling are often considered the best approach to monitor fish spawning migrations in rivers^1^. With the ongoing increasing use of DNA-based methods in the field of ecology, a new powerful approach has emerged for studies of aquatic ecosystems^7^. The investigation of aquatic species via environmental DNA (eDNA) – DNA fragments deposited into the water through faeces, mucus, or spawning products and extracted via precipitation or filtration from water samples – can be advantageous in many aspects^8^. eDNA analysis can be carried out with minimal disturbance to detect individual species via diagnostic end-point PCR or real-time qPCR^9,10^, or to assess whole fish communities via metabarcoding^11^. Furthermore, rare or invasive fish species are more likely to be detected via eDNA compared to conventional approaches^12,13^ and economic advantages such as lower costs^14^ and lower sampling efforts^15,16^ have been previously highlighted.

When eDNA is captured and analysed in riverine systems, downstream transport coupled with discharge and other environmental variables must be taken into account^8,17^. Transport is positively correlated with river size^17^ with maximum detection distances below 1 km in small streams^9,18^ and between 3 and 60 km in large rivers (discharge >3 m³/s)^17,19^. The transport distance is also influenced by the riverbed through the DNA retention properties of different substrate types^20,21^ and finally, increased discharge results in higher dilutions of eDNA^22^. Nevertheless, the eDNA signal was found to vanish within one day after the removal of source organisms from lotic systems^18,23^. Apart from monitoring fish communities in rivers, the potential of eDNA for evaluating fish migrations in these systems has been recently recognised and used to confirm the proper functioning of fish ladders^16^ and upstream migration of endangered sturgeons^24^. Besides the detection of fish species, there have also been attempts to relate eDNA concentrations to species abundance or biomass showing that the molecular approach does in general have the potential to reflect these parameters^25–27^. In rivers, eDNA copy number has been found to positively correlate with visual fish observations^28,29^, fish biomass in cages^18^, and electrofishing surveys^30,31^.

Despite these encouraging findings, eDNA is rarely used to monitor fish spawning migrations: for example, upstream migrating European anadromous shads were recently detected via eDNA^32^, and spawning activity of Australian Macquarie perch was estimated by comparing ratios of nuclear and mitochondrial eDNA^33^. Regarding the quantitative assessment of spawning migrations, case studies on Bigheaded carps, sockeye salmon and the sea lamprey find a positive correlation between adult fish numbers and eDNA signal strength^22,34,35^. However, the direct correlation between fish numbers, daily eDNA concentrations, and discharge has only been established recently for salmon migrations at an artificial weir^36^. Prior to this, semelparity, i.e. dead salmon releasing approximately ten times more eDNA compared to living individuals, partly obstructed the direct predictions of adult fish numbers^35^.

**Figure 1:**
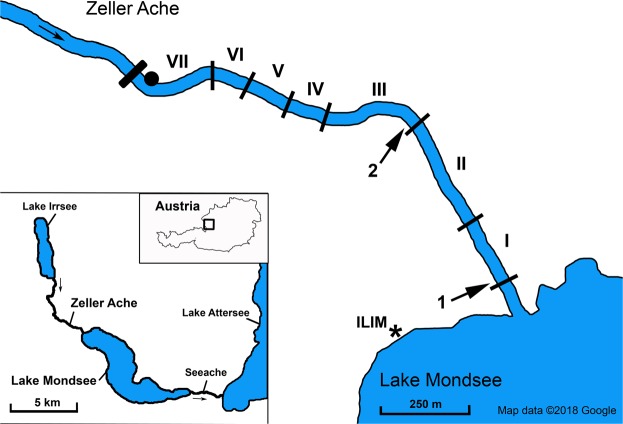
Overview of the Natura 2000 region “Mondsee-Attersee” (Austria) with the river Zeller Ache on the north-eastern tip of lake Mondsee; investigated river sections of the Zeller Ache: I: outflow-section, II: section with paved riverbed and bank, III: preferred spawning ground in previous years, IV and VI: restructured river sections, VII: most distant section from outflow with impassable dam on upstream end, V: non-accessible section (ca. 100 m) (see Supplementary Information 1 for detailed description of the river sections). Arrows indicate locations of eDNA water sampling and physicochemical measurements (point 1 and 2); the dot marks the location of the automated discharge measurement station. The asterisk shows the location of the Research Department for Limnology, of the University of Innsbruck (ILIM). This map was modified from Google Maps (Google Maps. Mondsee, Austria. Retrieved October 8, 2018, from https://goo.gl/maps/817sNygimuThE58t7 (n.d.)) using Adobe Photoshop C2 Version 9.0.

The aim of the present study was to evaluate the potential of eDNA for quantitative monitoring of the spawning migration patterns of the protected potamodromous and iteropoarous fish species *Alburnus mento* (Danube bleak), and the co-migrating species *Vimba vimba* (Vimba bream)^37^ in the Natura 2000 protected region “Mondsee-Attersee” (Austria, see Fig. 1). Artificially modified parts of the river bottom and banks (Supplementary Information 1) could hinder protected *A. mento* from reaching suitable spawning areas. Therefore, regular quantitative monitoring of migrating individuals is key to evaluate future population development. The two species were jointly counted every day throughout their spawning season and water samples for eDNA analyses were collected at the downstream end and in the middle of the examined river stretch. First, we evaluated the general relationship between fish counts and eDNA signals taking into account the influence of fish located upstream of the water sampling points and the mean daily discharge. Second, we tested if the two datasets follow the same pattern over time, i.e. show the same day-to-day fluctuations in the course of a spawning season. Finally, an eDNA-based time series model was tested for its potential to describe fish counts over time in order to assess the possibility of solely eDNA-based quantitative monitoring of spawning migrations in the future.

## Results

### eDNA signals, spawning migration, and fish counts

Weak eDNA signals (1.3–5.3 copies/µl extract) of both *A. mento* and *V. vimba* were detected via digital PCR between the 19^th^ April and 12^th^ May 2017, with first detections via endpoint PCR coupled with capillary electrophoresis (further on “CE-PCR”) on the latter day followed by the first visual observations and a steep increase in eDNA signals on 13^th^ May. The maximum number of *A. mento* and *V. vimba* individuals (further on “fish counts”) in the whole river was estimated as 8,625 on 17^th^ and 18^th^ May (see Materials and Methods for details on fish counts). At the upper end of the examined reach (section VII), less than 10 individuals were observed on three sampling days. Most spawning activity occurred at the river’s downstream end plus in the middle (sections I and III; Fig. 1). Generally, target eDNA concentrations were approx. twice as strong for *A. mento* (mean: 2,464 copies/µl extract ±5,747 SD; max: 39,473 copies/µl extract) compared to *V. vimba* (mean: 1,031 copies/µl extract ±2,176 SD; max: 15,525 copies/µl extract). This difference was not detectable in CE-PCR (*A. mento*: mean: 1.48 relative fluorescence units (RFU) ± 1.13 SD; max: 3.20 RFU; *V. vimba*: mean: 1.36 RFU ± 1.13 SD; max: 3.49 RFU).

### Relationship between eDNA signals and fish counts

**Figure 2:**
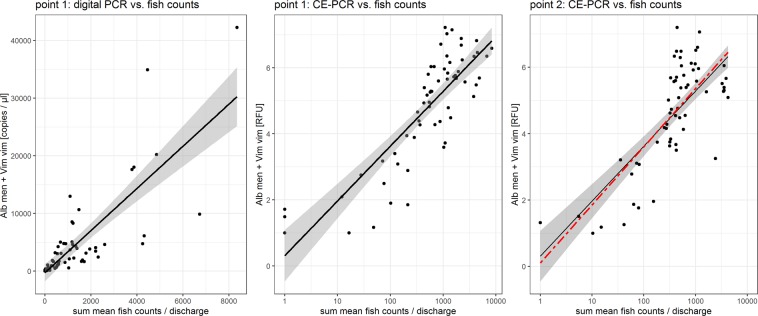
Relationship between eDNA concentrations/signals and discharge corrected fish counts. The left panel displays eDNA concentrations (copies/µl extract) obtained at sampling point 1 and discharge corrected fish counts of the whole river stretch plus model DD3. The middle panel shows RFUs obtained at sampling point 1 and discharge corrected fish counts of the whole river stretch combined with model EP3; 95% confidence areas of the models are depicted in grey. The right panel displays eDNA signals (RFU) obtained from sampling point 2 plotted against the sum of mean fish counts of section III and upstream (discharge corrected). The black line depicts EP3 fitted to the displayed data (R² = 0.58). The red, dashed line shows the optimum model obtained from this data (sub)set (EP4), which remains inside the confidence limits of model EP3 for the entire data range. X-axes of the middle and right panels are scaled logarithmically.

During the actual spawning migration (13^th^ May to 21^st^ July), eDNA signals (digital PCR and CE-PCR) from samples taken at the downstream end of the Zeller Ache were best described by a model including fish counts obtained from the whole examined river stretch (sections I to VII; 1.1 km) and the effect of mean daily discharge (model DD3: R² = 0.64; model EP3: R² = 0.71; Tables 1 and 2, and Fig. 2). Fish counts were positively related to target eDNA signals obtained via the two molecular approaches (digital PCR: parameter estimate = 3.66; P < 0.001; CE-PCR: parameter estimate = 0.72; P < 0.001; from DD3 and EP3 in Table 3). The optimum model derived from fish counts in section III and upstream and water samples taken at point 2, i.e. the downstream end of section III (Table 3: EP4; R² = 0.58; based on CE-PCR and the discharge corrected sum of mean fish counts), did not differ significantly from the best performing model derived for the whole river stretch (EP3), which was fitted onto this dataset (R² = 0.58; Fig. 2).

**Table 1:** The set of candidate models used to investigate, which mean fish counts (only lowest river section (ZA I) or sum of all sections (ZA)) provide the best fit to eDNA signals obtained at point 1 and if the inclusion of mean daily discharge significantly improves model fit.

Model #	Model description
DD1	copies/µl = mean fish counts at ZA I
DD2	copies/µl = mean fish counts whole ZA
DD3	copies/µl = mean fish counts whole ZA/discharge
EP1	RFU = ln(mean fish counts at ZA I)
EP2	RFU = ln(mean fish counts whole ZA)
EP3	RFU = ln(mean fish counts whole ZA/discharge)

Models DD1 to DD3 include eDNA concentration obtained from digital PCR (copies/µl extract) and were compared to each other; models EP1 to EP3 include eDNA signal strength obtained from CE-PCR (RFU) and were compared to each other.

**Table 2:** Results of the ordinal ΔAICc-based ranking of model performance carried out separately for digital PCR (DD1 to DD3) and CE-PCR (EP1 to EP3) models.

Model #	K	AICc	ΔAICc	ω	R²
DD3	3	1246.76	0.00	0.54	0.64
DD1	3	1248.42	1.66	0.24	0.63
DD2	3	1248.57	1.81	0.22	0.63
EP3	3	173.53	0.00	1.00	0.71
EP2	3	189.61	16.08	0.00	0.62
EP1	3	212.53	39.00	0.00	0.45

K (number of estimable parameters), AICc (second-order variant of Akaike’s Information Criterion), ΔAICc (AICc differences), ω (Akaike weight), and R² are reported with models ordered from high to low weight.

**Table 3:** Summary of the models best describing the relationship between eDNA signals and fish counts during the actual spawning migration.

Model #	Predictor variable	Parameter estimate	SE	Lower 95% CI	Upper 95% CI	t-value	p-value
DD3	intercept	−272.72	754.62	−1,751.75	1,206.32	−0.36	0.72
	mean fish counts whole ZA / discharge	3.66	0.35	2.96	4.35	10.32	<0.001
EP3	intercept	0.31	0.39	−0.45	1.08	0.80	0.42
	ln(mean fish counts whole ZA / discharge)	0.72	0.06	0.60	0.84	12.08	<0.001
EP4	intercept	0.10	0.52	−0.91	1.12	0.20	0.84
	ln(mean fish counts ZAIII & upstream / discharge)	0.76	0.08	0.59	0.93	8.96	<0.001

Model DD3 (digital PCR data) and model EP3 (CE-PCR data) are based on point 1 eDNA samples and fish counts in the entire river stretch; model EP4 (CE-PCR) is based on data from section III and upstream. Parameter estimates, standard errors (SE), lower and upper confidence intervals, t-values and p-values are displayed.

### Characteristics of target eDNA concentration and fish counts over time

**Figure 3:**
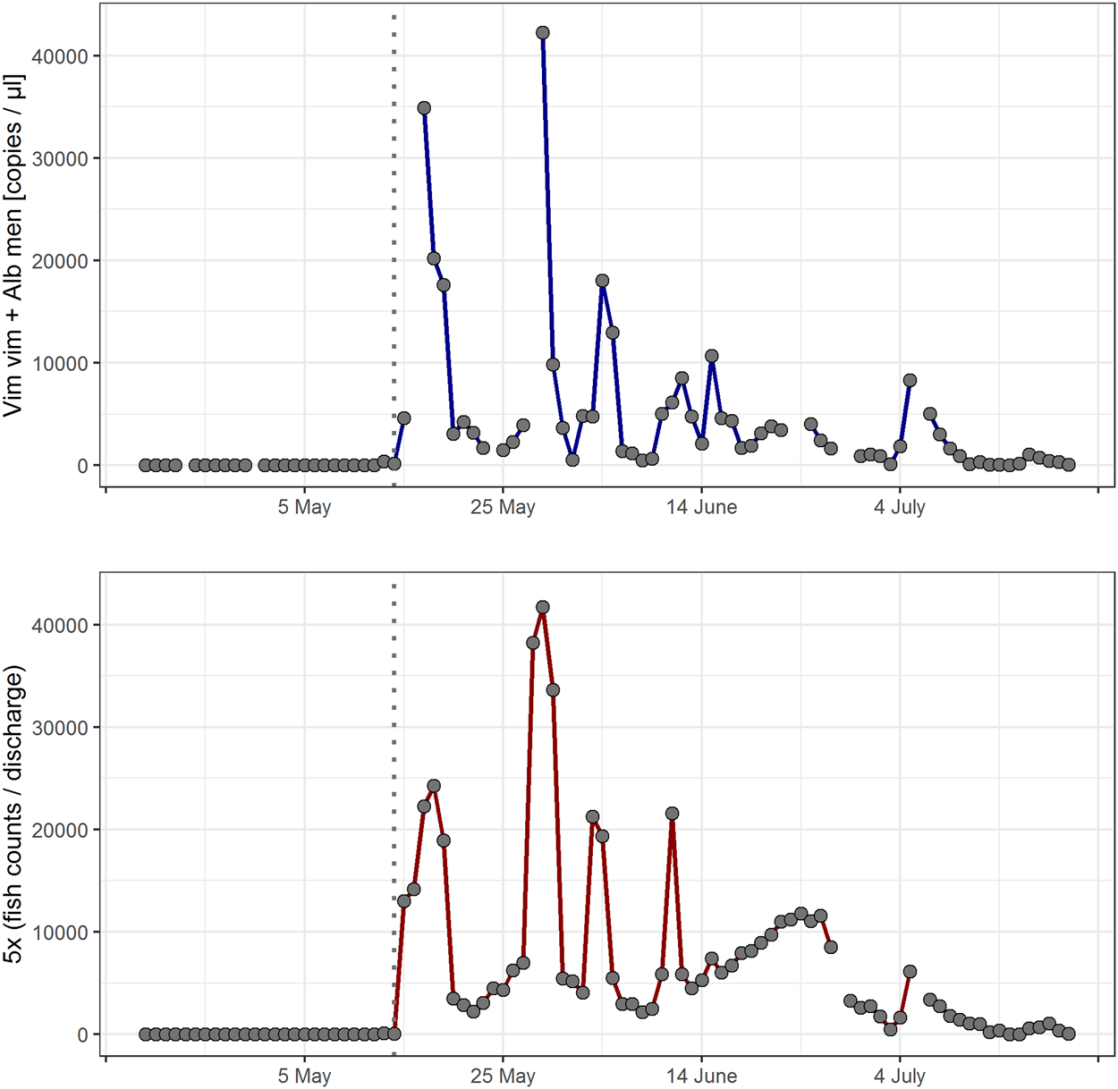
Time series of target eDNA concentration obtained via digital PCR (upper panel) and fish counts (lower panel). To obtain comparable amplitudes between the two time series, fish counts were summed up for the whole river stretch and divided by discharge; the resulting values were thereafter individually multiplied by five. Gaps denote days without measurements, which were filled by averaging neighbouring values for time series analysis. The dotted lines indicate the start of the actual spawning season on 13^th^ May characterised by first visual observations and a steep increase in eDNA signals.

The analysis of both target eDNA concentrations (digital PCR) and fish counts over time (see Materials and Methods for data transformations and included discharge effect) showed that the two time series were highly correlated (*r*_*s*_ = 0.91) across the whole investigation period (Fig. 3). Cross-correlation between eDNA signals and fish counts was highest when the two time series were not lagged (cross-correlation = 0.82; but see Supplementary Fig. 1 for cross-correlations at different lags), meaning that simultaneous day-to-day changes showed the highest similarity between the two datasets. The observed cross-correlation was significant, as it lay outside the 95% confidence interval generated from 1,000 cross-correlation values from random sorting of the two time series. A closer investigation of daily trends showed that eDNA concentrations and fish counts were changing along the same direction on 62 out of 70 days (both eDNA and fish counts >0), which was significantly different from a 1:1 ratio (Chi² = 22.69; P < 0.001). Furthermore, both time series showed significant auto-correlation i.e. influence of previous values (eDNA: Chi² = 39.26, P < 0.001; fish counts: Chi² = 50.67, P < 0.001).

### Time series modelling

**Figure 4:**
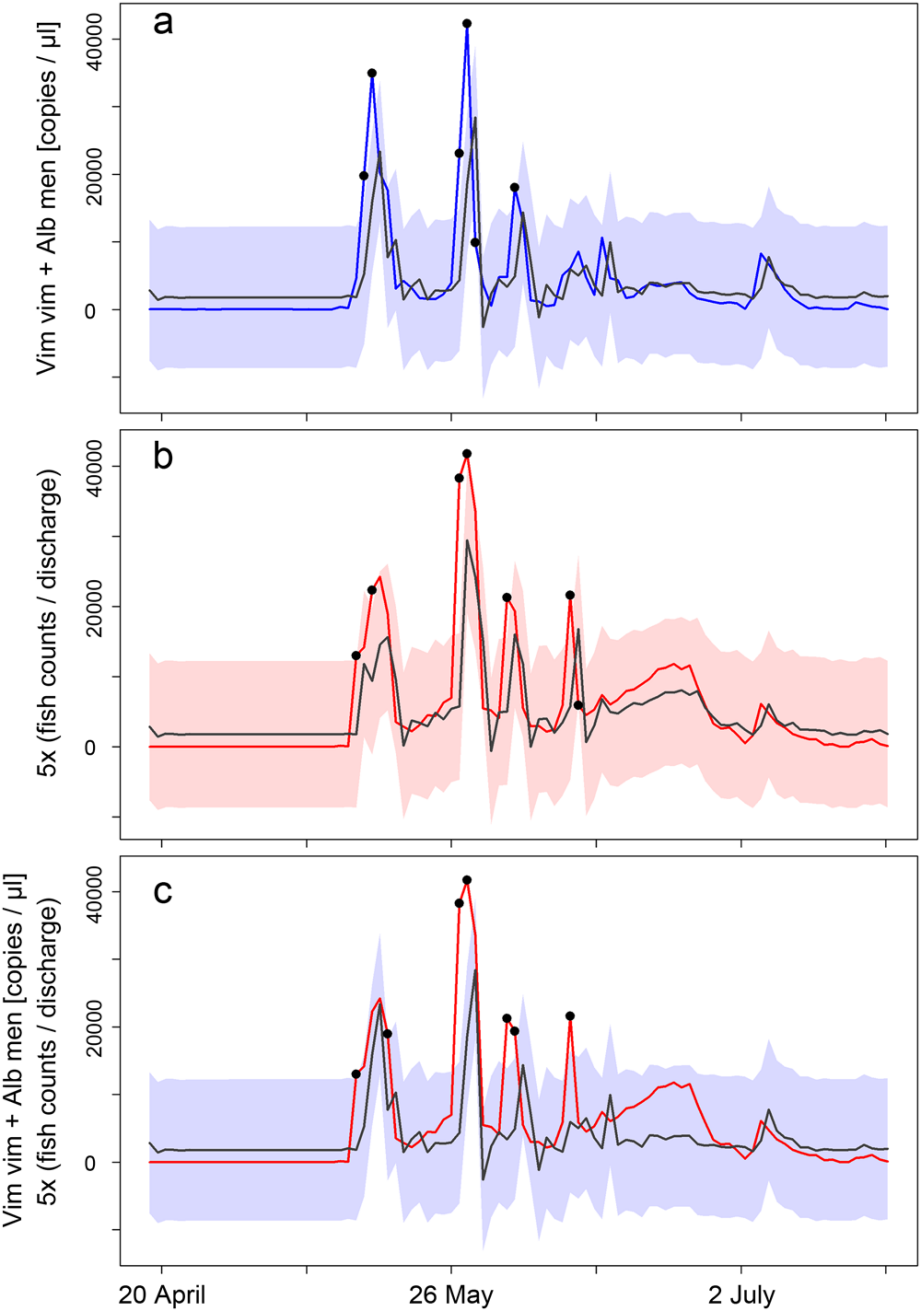
Time series models in comparison to measured target eDNA concentration and fish counts. (**a**) The ARIMA (2, 0, 0)-modelled eDNA concentration (grey line) and 95% confidence area (blue shading) in comparison to measured eDNA concentration (blue line). (**b**) Modelled fish counts using the same ARIMA model structure (grey line; red shading for 95% confidence area) in comparison to the actual fish counts (red line). (**c**) ARIMA-modelled eDNA concentrations (grey line and blue shading) in comparison to actual fish counts (red line). Values outside the 95% confidence area are marked as black dots, eDNA concentration is based on the sum of *Alburnus mento* and *Vimba vimba* (copies/µl extract) from point 1 samples, and fish counts are the sum of mean counts from all river sections divided by discharge; to enable direct comparison between time series, these values were additionally multiplied by five.

To evaluate the potential of solely eDNA-based monitoring for spawning migrations, the relationship between target eDNA concentration (digital PCR) and fish counts was evaluated via time series modelling in three steps: first, we determined the optimum ARIMA model for describing eDNA concentrations. Second, we tested whether the same ARIMA model structure (order, differencing, and order of the moving-average model) was suitable to describe the fish count data. Third, we evaluated whether actual daily fish counts could be described by the optimum eDNA-based ARIMA model. eDNA concentrations were best described via an ARIMA model ((2, 0, 0); AIC = 1,885.45) taking into account two lagged values, i.e. eDNA signals from the two previous days (order). This model was further characterised by a mean average error of 2,930.86 and a first-order auto-correlation coefficient of 0.0004 (Table 4). It presented a good fit (based on standardised residuals and non-significant Ljung-Box statistics at lags 1 to 10) to the measured eDNA concentrations. These lay outside the 95% confidence area only on six days; five were characterised by large signal amplitudes and one by rapid change in eDNA concentration (Fig. 4a). The ARIMA model structure was subsequently applied to the fish count time series to test whether target eDNA concentration and fish counts could be described in the same way. Generally, a sub-optimal fit of the ARIMA model structure to fish count data was indicated by two significant Ljung-Box statistics (lags 4 and 6), a higher first-order auto-correlation coefficient of 0.14, and a higher AIC of 1,890.64. Nevertheless, visual inspection (Fig. 4b) showed a good fit between modelled and measured fish counts: fish count values lay outside the 95% confidence area only on seven days (six at large signal amplitudes; one at a drastic day-to-day change; Fig. 4b). Finally, the eDNA-based ARIMA model was compared directly to the obtained fish counts: the latter lay inside the 95% confidence area of the model except for seven days, which were all characterised by high fish counts (Fig. 4c).

**Table 4:** The ARIMA model ((2, 0, 0); AIC = 1,885.45) best describing target eDNA concentrations (digital PCR) including parameter estimates, standard errors (SE), lower and upper confidence intervals, z-values and p-values for the two auto-regressive components and the intercept.

Component	Parameter estimate	SE	Lower 95% CI	Upper 95% CI	z-value	p-value
intercept	3,718.37	1,185.84	1,394.17	6,042.57	3.14	0.002
AR 1	0.79	0.10	0.59	0.98	9.35	<0.001
AR 2	−0.24	0.10	−0.43	−0.04	−6.62	0.02

## Discussion

Our study substantiates the applicability of eDNA for the investigation of potamodromous fish spawning migrations not only in terms of presence/absence data, but also concerning quantitative approximations. As expected, there was a positive correlation between eDNA concentrations and visual fish counts including parallel changes in both parameters on a day-to-day basis. Besides fish individuals present in close proximity to the water sampling points, fish from further upstream (up to 1.1 km) significantly contributed to the detected eDNA signals. Increased mean daily discharge negatively influenced eDNA concentration and model fit was substantially improved when it was taken into account. The relationship between discharge corrected fish counts of the whole river stretch and eDNA signals (CE-PCR-based) from its downstream end could also describe the situation in the upper half of the river. Concerning the changes of both eDNA concentrations and fish counts during the spawning season, the same time series model structure was capable to depict both eDNA concentrations and fish counts within the 95% confidence area for 94% of the examination period. Additionally, the eDNA-based time series model was suitable to describe fish counts directly during the whole field study, except for days with highest fish numbers, on which the model slightly underestimated the height of the fish count spikes. This demonstrates the high potential of quantitative eDNA analysis to monitor spawning migrations of potamodromous fish species under known discharge conditions.

The general positive correlation between eDNA signals and the number of fish commencing spawning migrations confirmed results of previous studies on Bigheaded carps, sockeye salmon, and sea lampreys^22,34,35^. As the present study used visual fish counts, it was possible to relate eDNA signals to the whole adult *A. mento* and *V. vimba* population present in the examined river stretch similar to salmon counts at an artificial weir^36^. Additionally, spawning has no lethal effect on the examined iteroparous species *A. mento* and *V. vimba*^37,38^ omitting the effect of dead individuals, which can significantly influence eDNA levels^35^. Albeit a positive correlation between visual observations and eDNA signals has been previously observed^28,39^, factors such as discharge and the distribution of target organisms in the river need to be considered to correctly describe this relationship^35,36,40^. Our model comparison indicated a substantial contribution of fish individuals from further upstream (up to 1.1 km) to target eDNA concentrations at the downstream end of the examined reach. The role of eDNA deposition could not be directly assessed in the Zeller Ache based on the obtained measurements and during preliminary data analysis only a very small sedimentation rate of 1% per 100 m (compared to 50% within 500–1,000 m based on previous studies^9,17^) showed a favourable effect on model fit (Supplementary Method 1). Hence, it was deemed unjustifiable to include eDNA sedimentation in the modelling process at all. Explanations for this are provided by the structure of the riverbed and the distribution of migrating fish individuals: rows of large boulders in sections IV and VI induced high turbulence and partially coarse substrate in sections I, III, and VII hindered eDNA deposition^21^. Additionally, the riverbed in section II (~250 m), connecting the two sections with highest fish counts, was fully paved, which potentially blocks the entire eDNA deposition process (Supplementary Information 1).

Apart from influencing downstream transport distance, higher discharge potentially leads to lower eDNA signals via dilution^18,36^ and was found to be critical for a meaningful quantitative interpretation of eDNA signals emitted from migrating salmon^36^. Potamodromous fish/cyprinids usually migrate only a few kilometres to suitable spawning grounds in medium sized rivers, which are characterised by high discharge fluctuations during spring and summer^3^. In our study, the mean daily discharge of the Zeller Ache varied tenfold during the spawning period from 0.25 to 2.5 m³/s. Therefore, the necessary inclusion of discharge in the models best describing the relation between fish counts and eDNA signals was not surprising. To evaluate the applicability of a model best describing the situation in the Zeller Ache to similar spawning migrations, its performance was tested on a subsection of the river (section III and upstream) based on independent eDNA samples and ~60% of the total fish counts. The model obtained from the whole river stretch did not differ significantly from the optimum model estimated for this “subset” (Fig. 2). Albeit fish counts in the comparison were not independent, this indicates that the model is applicable in similar situations concerning fish counts, discharge, and size of a spawning reach.

Other factors influencing eDNA decomposition and leading to reduced detectability include higher water temperatures, UV-B radiation, acidic pH, and water sample storage prior to filtration^41–43^. Fortunately, fast downstream transport and the proximity between migrating fish and water sampling points in our study should have minimised the influence of the above mentioned factors, which exhibit negative affects after hours or days^23,42^. Water sample storage was previously found to negatively affect eDNA persistence^43,44^, but the close proximity between sampling points and the laboratory used for filtration (Fig. 1) enabled sample processing within ~1.5 h. Additionally, water samples were always taken in the same order and the time until filtration remained constant throughout the study. Hence, eventual DNA decomposition prior to filtering can be considered similar for all samples.

In samples taken during the two weeks prior to the start of the spawning migration, very low levels of *A. mento* and *V. vimba* eDNA were detected via digital PCR (1.3–5.3 copies/µl extract). These could stem either from individual adult fish probing the lowest section of the Zeller Ache before the onset of the actual spawning migration or from populations with unknown spawning patterns in Lake Irrsee (Fig. 1) on the upper end of the Zeller Ache^45^. eDNA signals from upstream are, however, unlikely to have a general influence on the obtained data as the upper end of the examined spawning reach and Irrsee are separated by ~6.3 km and 16 impassable transverse structures. Additionally, low flow velocities at the outflow of Irrsee render this short part of the river inexpedient for spawning of *A. mento* and *V. Vimba*^45^.

Time series analysis of eDNA concentrations obtained via digital PCR and fish counts showed that both datasets follow the same pattern during the whole examination period including similar day-to-day changes and influence of values measured on two previous days. The eDNA-based ARIMA model onlystruggled to incorporate the highest fish counts within the 95% confidence area (Fig. 4c). This discrepancy could be caused by the categorical estimates of fish numbers (see Materials and Methods). To minimise subjective perception, two skilled experts did the estimations simultaneously and classifying fish abundance in six categories was easily possible. However, category size increased with increasing fish numbers and estimations were less precise at higher numbers (Fig. 2 left panel, Fig. 4c). Despite this limitation, we considered visual observations most appropriate for fish counts in the examined situation^1^, especially since the size of the Zeller Ache (wetted width: ~5 m; depth: 0.2–1.5 m) coupled with low turbidity throughout the spawning season enabled reliable fish counts. On the four days when the water was not completely clear, turbidity was still low enough to estimate fish numbers. Nevertheless, *A. mento* and *V. vimba* were hardly distinguishable from outside of the water, resulting in joint fish counts and the potential for bias in case the two species release eDNA at different rates. As both are cyprinids of the same average size (~25 cm), inhabit Alpine foreland lakes, and display the same activity level during spawning^38^, species-specific eDNA emission rates are an unlikely bias in the present study. Furthermore, *A. mento* and *V. vimba* constitute over 90% of all migrating fish during spring and early summer in the Zeller Ache^37^, making other species, such as the rheophile *Salmo trutta*, negligible in the examined situation. Additionally, the third and fourth most abundant species (*Rutilus meidingeri* and *Squalius cephalus*) could be excluded due to their much larger size and earlier spawning of the former^37^. Finally, fish present in the unobservable section V could have biased fish counts, but due to its short length (100 m) and comparably low fish counts in section IV below (16% of total counts) the influence should be minute.

Throughout the whole investigation period, it was not possible to quantify actual spawning activity. Hence, milt, eggs, and hatched larvae potentially affect the correlation between eDNA concentrations and fish counts. The influence of milt and eggs should be minute in the examined situation as spermatozoa primarily contain nuclear DNA^33^ and milt degrades within hours in mesocosms^46^. Furthermore, the eggs of *A. mento* and *V. vimba* stick to the substrate^4^ and the number of drifting eggs could so far not be correlated with eDNA signal strength^34^. Hatched fish larvae are readily detectable via eDNA as demonstrated for burrowing lampreys^47^ and potentially influence species-specific eDNA signals. However, the larvae of *A. mento* are known to migrate downstream soon after hatching^38^ and high flow velocities prevailing in the river should lead to a fast downstream transport. When comparing eDNA concentrations to fish counts, additional peaks or inflated peak heights induced by milt, eggs, and larvae were not detected. Only the height of two small peaks in the second half of the examination period points to the influence of hatched larvae, as fish counts did not follow this pattern or were less high on these two occasions.

We applied two molecular detection systems based on species-specific primers and digital PCR or CE-PCR, the first permitting absolute quantification of target DNA copy number^48^, the second enabling semi-quantitative estimations (Supplementary Method 2). The applied primers amplify short fragments (≤200 bp) and were subjected to extensive specificity testing to exclude the possibility of non-target DNA amplification in central Europe at the reported PCR conditions^49^ (Supplementary Method 2). A direct comparison between digital PCR and CE-PCR confirmed the higher sensitivity of digital PCR and showed reliable positive CE-PCR detections from more than 25 (*V. vimba*) and 42 (*A. mento*) copies per µl extract, which was sufficient for the amplification of eDNA stemming from ~11 fish individuals present in and above section III (600 m) at 2.44 m³/s discharge. In practice, this sensitivity difference only played a role for samples taken before the onset of the actual spawning migration, some of which tested positive in digital PCR but not in CE-PCR. The high eDNA levels detected during the actual spawning migration also resulted in a saturation effect in CE-PCR^50^ and similar signal strengths for *A. mento* and *V. vimba* (Supplementary Method 2), whilst absolute eDNA concentrations were higher for *A. mento*, corresponding to fish trap data collected in the Zeller Ache in 2010^37^. Concerning the general relation between fish counts and eDNA signals, the saturated CE-PCR in this special case coincided with less precise fish counts in higher categories and led to smaller dispersion in CE-PCR-based models (Fig. 2).

Two factors warranting additional discussion of water sample processing and molecular analysis are DNA cross-contamination and PCR inhibition, both of which frequently affect eDNA-based studies^9,44^. We down-corrected eDNA signals of samples affected by cross-contamination via filtering equipment by the respective RFUs or copy numbers of the six filtration controls, all of which showed weak signals (<0.23 RFU; <1.32 copies/µl) in comparison to affected field samples (>2.1 RFU; >1,100 copies/µl). Thus, the influence of DNA contamination was likely removed from the data set without completely dropping affected samples and avoiding data gaps in time series analysis. The DNA contaminations most likely stem from the water filtration process, as negative controls added during DNA extraction and PCRs all resulted negative. These slight cross-contaminations occurred despite rigorous cleaning procedures (see Materials and Methods). However, only sodium hypochlorite concentrations of 3.6% were available contrasting to the 6% commonly used in US laboratories^44^. Future eDNA studies would definitely benefit from the use of encapsulated filters and higher concentrations of sodium hypochlorite and/or longer incubation times^44,51^. With regard to PCR inhibition, additional clean up steps were not applied during sample processing. Nevertheless, BSA, known to reduce PCR inhibition^52^, was included in all CE-PCR assays and the applied extraction method performs well with problematic samples such as plants or tissues with a high lipid content^53^. Finally, plant litter and associated humic substances, previously found to hinder successful amplification of eDNA^18,52^, were not present throughout the study period.

Our study demonstrates the positive correlation between eDNA concentration and fish counts on a day-to-day basis during spawning migrations of potamodromous fish. In the medium-sized Zeller Ache and its 1.1 km long spawning reach, downstream eDNA signals were defined by fish individuals present in this whole stretch and changes in the daily discharge significantly affected the relationship between eDNA levels and fish counts, indicating a strong dilution effect associated with increased water run-off. Finally, the eDNA-based time series model allowed approximating fish counts for almost the entire examination period. As such, our findings highlight the capability of eDNA beyond simple presence/absence investigations towards efficient and informative monitoring of dynamic aquatic processes and support future conservation efforts within the scope of the water framework directive and habitats directive of the EU.

## Materials and Methods

### Study site

The river Zeller Ache is the main spawning ground of *A. mento* and *V. vimba*, two spring- and early summer-spawning cyprinid species migrating from May until the end of July out of the Upper-Austrian lake Mondsee (N47°48′56.8″ E13°22′54.8″) upstream into the river Zeller Ache for reproduction until their path is blocked by an impassable dam after around 1.1 km (Fig. 1)^37^. The Zeller Ache (38.3 km² catchment area) has a mean discharge of 1.98 m³/s and flows ~7.4 km from lake Irrsee into Mondsee with a height drop of 73 m^54^. The wetted width in the examined reach was ~5 m and depth ranged from 0.2 to 1.5 m. Due to the proximity to villages and streets, it is heavily modified with stone built river beds or banks and numerous transverse structures^45^ leading to higher flow velocities and fewer opportunities to rest (Supplementary Information 1). All field work along the Zeller Ache was carried out between 19^th^ April and 21^st^ July 2017 (94 days) between the inflow into Mondsee and an impassable dam around 1.1 km upstream. This reach was separated into seven sections based on structural differences of the riverbed (e.g. natural or artificially paved river bottom, known or unknown use for spawning in former seasons; Fig. 1, Supplementary Information 1). River section V (100 m) could not be accessed as it is completely bounded by private properties and buildings.

### Visual fish counts and environmental parameters

In each section, upstream migrating *A. mento* and *V. vimba* were visually counted on a daily basis from the riverside (if accessible) and/or from bridges (except days 5, 71, and 79). Observations were carried out by two skilled experts moving upstream within approx. one hour and mostly in the morning between 8 am and 12 noon. Per section, only five to 10 min were used for fish estimations to exclude double counts by upstream migrating individuals. Fish were counted categorically in each section (Cat. 1 = 1–5 individuals, Cat. 2 = 5–10 ind., Cat. 3 = 10–50 ind., Cat. 4 = 50–100 ind., Cat. 5 = 100–500 ind., Cat. 6 ≥ 500 ind.) whereby *A. mento* and *V. vimba* were grouped together as these two species are hard to distinguish from outside of the waterbody. Due to the high numbers and movement of the migrating fish, it was not possible to obtain exact fish counts from visual observations. To account for larger estimate errors associated with increasing individual numbers, it was furthermore necessary to increase category sizes with increasing numbers.

### eDNA sampling and processing

Water for eDNA analysis was taken daily (except days 5, 12, 28, 36, 40, 66, 67, 71, 72, and 79) in wide neck bottles with a volume of 2 L and an opening diameter of 50 mm. These were treated with chlorine bleach (3.6 g sodium hypochlorite per 100 g liquid) overnight and thoroughly washed with fish-DNA-free tap water prior to use. One water sample was taken at point 1 (downstream end of section I) and point 2 (downstream end of section III; Fig. 1) in the middle of the river at 5–10 cm depth and bottles were plunged at arm length against the current. Fresh, DNA-free gloves were used for handling of each water sample, which were stored and transported within two hours to the Research Department for Limnology (Mondsee, Austria; Fig. 1) for further processing. After water sampling, the water temperature, pH, oxygen saturation and electric conductivity were measured with a portable multi meter HQ40d (HACH) at point 1 and 2. Water temperature ranged from 6.8 to 24 °C with 13 °C at the beginning of migration activity; pH ranged between 8.23 and 8.81. Due to time constraints, turbidity was only visually classified into “low”, “medium”, and “high”, and found to be low to medium with clear visibility of the river bottom except for days 20, 21, and 88 (before the beginning and at the end of the spawning migration). Data on the daily mean discharge (m³/s) during the investigation period were collected at section VII (Fig. 1) at a fixed measurement station and provided by the department of surface water management Upper Austria. Discharge ranged from 0.25 to 4.79 m³/s (thunderstorm induced peak on the 9^th^ May; day 22) and never exceeded 0.5 m³/s between 18^th^ June and 12^th^ July. Above the measurement point, the Zeller Ache has no inflow until the upper end of section VII; below the measurement point, the river has two small inflows with approximate widths of 0.5 and 1.5 m; their influence was neglected for all further calculations.

On arrival at the institute, samples were vacuum-filtered through 47 mm glass fibre filters (1.2 µm mesh width; Whatman GF/C) using a water-jet pump (BRAND). Between samples, the filter equipment was decontaminated for about 15 min in chlorine bleach (0.36 g sodium hypochlorite per 100 g liquid) and thoroughly washed with fish-DNA-free tap water. On every second to third day, a negative control (2 L MilliQ water) was filtered before and after the field samples of the respective day. Filters were folded with DNA-free, flamed forceps and stored separately in 1.5 ml reaction tubes at −20 °C until DNA extraction. Every day, lab surfaces were cleaned with bleach and ethanol (70%) prior to filtering and DNA-free gloves were worn at all times. For further processing, filters were transferred in a cooling box to a clean room laboratory at the University of Innsbruck (Austria) within three hours. Lysis, DNA extraction, amplification, visualisation, and quantification using digital PCR and CE-PCR were carried out there (Supplementary Method 2). This was accompanied by a test with dilution series confirming the suitability of the latter approach for semi-quantitative estimations of target DNA in PCR. Additionally, absolute target DNA concentrations (digital PCR) and eDNA signal strength (CE-PCR) were directly compared for field samples obtained from point 1 (Supplementary Method 2).

### Analysis

All statistical analysis and calculations were carried out with R (R Development Core Team 2017) using packages and functions described in Supplementary Method 3. To make fish counts comparable with the obtained eDNA signals, the mean number of fish of the respective categories were used (e.g. mean of Cat 1 (1–5 individuals) = 2.5). For Cat 6 (≥500 individuals) a maximum of 5,000 fish was estimated resulting in a mean value of 2,750 individuals. Fish counts of *A. mento* and *V. vimba* could not be carried out separately, hence, the eDNA concentrations (digital PCR) and signal strengths (obtained via CE-PCR and measured in RFUs) of *A. mento* and *V. vimba* were added up.

The absolute quantification (target DNA copies/µl extract) resulting from digital PCR permits the direct comparison of eDNA concentration to fish counts via linear models. To enable this comparison for eDNA signal strength obtained from CE-PCR, logarithmic models were necessary. For these, both independent (mean fish counts) and dependent (eDNA signal) variables were elevated by 1. Only data obtained between the 13^th^ May and 21^st^ July during the actual spawning migration of *A. mento* and *V. vimba* were used for model comparison to remove the bias of zero values and extremely low values obtained before the actual onset of the spawning migration. We compared different models (Table 1) for their descriptive performance based on ordinal ranking of the AICc, ∆AICc, and the weights of the AICc values^55^. For both molecular methods (digital PCR and CE-PCR), three models were compared using the same combinations of mean fish counts and discharge to explain eDNA signals at sampling point 1 (Table 1). In models DD1 and EP1, only fish counts from the most downstream section of the Zeller Ache (closest to point 1) were used. Models DD2 and EP2 included the sum of mean fish counts from all observed river sections. In models DD3 and EP3, mean fish counts from all river sections were additionally divided by the mean daily discharge. We refrained from the inclusion of a distance effect, after initial data analysis (Supplementary Method 1). In a next step, an additional logarithmic model (EP4) was calculated based on CE-PCR derived eDNA signals at sampling point 2 and the sum of mean fish counts from sections III and upstream (60% of total fish counted) divided by the daily discharge. The fit to this data “subset” was compared between model EP4 and the best performing model from the ordinal ranking.

To compare fish counts and target eDNA concentrations (digital PCR) in time series analysis, it was necessary to transform fish counts in a way to obtain similar fluctuation range and scaling for both data sets. Therefore, the value obtained by dividing mean fish counts by mean daily discharge was additionally multiplied by five. For missing values in both series, approximations were calculated using the mean of the previous and following value. At first, correlation and cross-correlation between the two time series was examined by calculating the Spearman correlation *r*_*s*_ and the normalised cross correlation. To test whether the obtained cross-correlation was significant, the fish counts and associated target eDNA concentrations were 1,000 times randomly sorted, the maximum correlation stored for each of the calculations, and a 95% confidence interval calculated (lower bound: 0.24; upper bound: 0.25). Each series was also separately tested for auto-correlation using the “Ljung-Box”-test. In a next step, we explored if fish counts and eDNA concentration change along the same direction from day to day: days without fish counts and eDNA signals were excluded from this analysis. To model eDNA concentration over time, an ARIMA model was calculated. Model fit was examined based on standardised residuals, auto-correlation, Ljung-Box statistic and the confidence intervals of coefficients. Finally, the obtained ARIMA-model was used to model fish counts; then, fit between modelled and measured eDNA signals and fish counts was compared by determining whether the actual values were located within the confidence area of the modelled time series.

## Supplementary information

Supplementary Information

## Data Availability

All data on fish counts, eDNA analysis, and abiotic parameters are available on Figshare; 10.6084/m9.figshare.9868193.v1.
