# Supplementary material for: Monitoring spawning migrations of potamodromous fish species via eDNA
Source: Sci Rep. 2019 Oct 28;9:15388. doi: 10.1038/s41598-019-51398-0 (PMC6817844; doi:10.1038/s41598-019-51398-0)
Supplement: Supplementary file 1 — Supplementary Information [file 41598_2019_51398_MOESM1_ESM.pdf]

Monitoring spawning migrations of potamodromous fish species via eDNA

Electronic Supplementary Material

Bettina Thalinger[2*], Elisabeth Wolf[1, 2*], Michael Traugott[2], Josef Wanzenböck[1]

*contributed equally to this paper

[1] Research Department for Limnology, University of Innsbruck, Austria

[2] Department of Ecology, University of Innsbruck, Austria

List of Supplementary Information, Methods, and Figures

Supplementary Information 1: Description of investigated river sections of the Zeller Ache

Supplementary Figure 1: Cross-correlation between eDNA signals and fish counts

Supplementary Method 1: Evaluation of eDNA deposition

Supplementary Method 2: Filter processing, DNA extraction, amplification, visualisation, and quantification

Supplementary Method 3: Packages and functions used for data analyses in R

Supplementary Information 1: Description of investigated river sections of the Zeller Ache

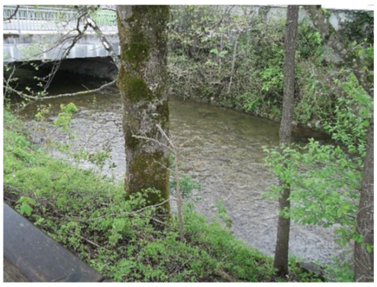

I) Section closest to Mondsee (length: ~ 170 m): This section includes approximately 30 m of suitable spawning conditions[1] and was observed between a wooden bridge (looking downstream and upstream) and walking along the river bank up to the first road bridge (Atterseestraße, again looking downstream and upstream as far as the observers' visual range).

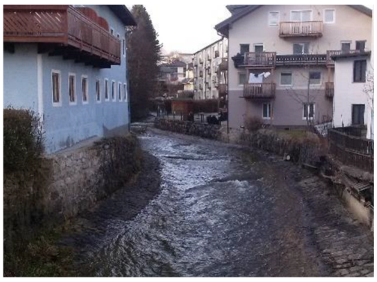

II) Intensively regulated section (~ 270 m): This section leads through the urban area of the city Mondsee and is strongly regulated by means of stone-built riverbeds and banks leading to higher flow velocities and few possibilities for migrating fish to rest. Transversely fixed tree trunks were installed about 20 years ago to improve the conditions for migrating fish, which was confirmed by visual observations of the species' reactions to these difficult conditions.

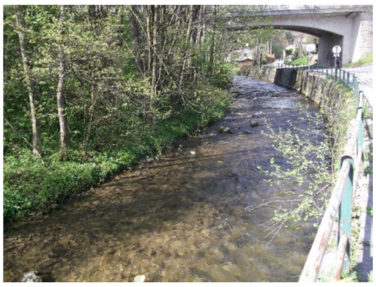

III) Main former spawning ground (~ 220 m): This section is known to be highly frequented by *Alburnus mento* and *Vimba vimba* during spawning migrations in previous years. It is less regulated than the section before and characterised by riverbank vegetation on at least one side of the river and by a less silted and more natural riverbed.

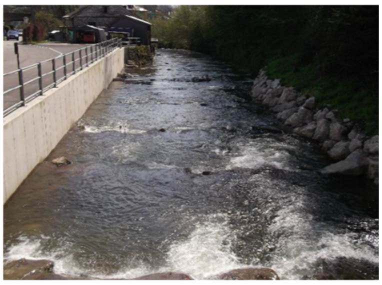

IV) Section of dam removal 1 (~ 70 m): According to the responsible consultant bureau (Schauer and Gumpinger), the dimensions of this construction are 12 basins each 7 m wide and 5 m long with 13 cm vertical drop between the basins. Basins are separated by rows of large boulders, leading to increased turbulence. In the basins themselves, which are approximately between 0.5 to 1 m deep, the current is more natural and less laminar than in sections further downstream.

V) *No picture as the whole section is closed off by private properties and buildings and not observable from outside the river.*

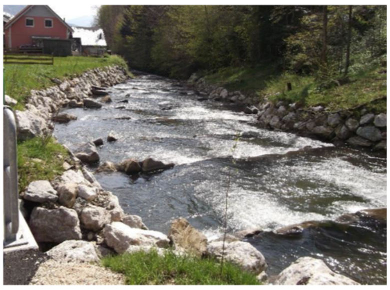

VI) Section of dam removal 2 (~ 80 m): The dimensions of this new construction are 18 basins, each 11 m wide and 3.5 m long with a maximal vertical drop of 13 cm. Further characteristics are the same as described for section IV.

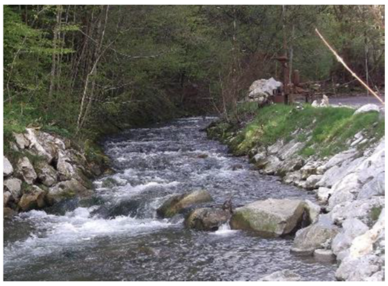

VII) Located between reconstruction 2 and an impassable dam (~ 200 m): It was not possible to observe this section from outside the river over the whole stretch as the riverside is partly closed off by private properties and buildings. The fixed discharge measurement station is located in this section.

Supplementary Figure 1: Cross-correlation between eDNA concentration and fish counts

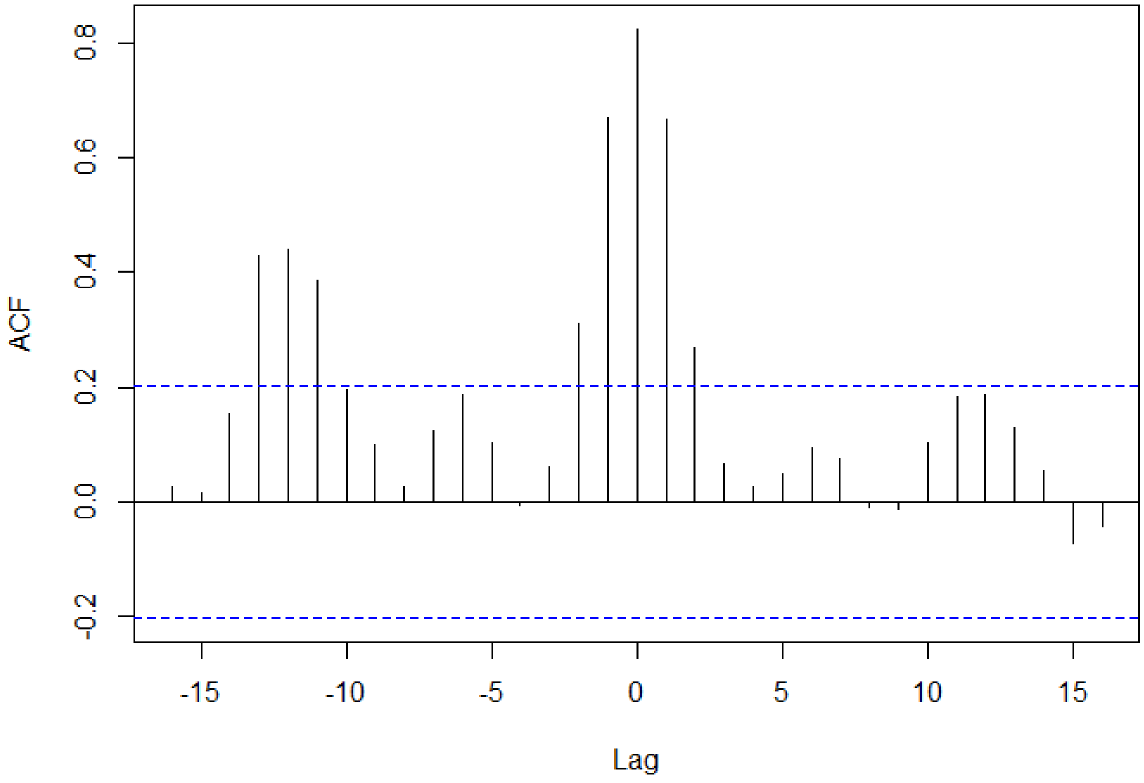

Supplementary Figure 1: Cross-correlation of eDNA signals (copies / µl extract) and the sum of mean fish counts divided by daily discharge, before multiplying the resulting values individually by five. The highest cross-correlation between the two time series was observed at a lag of zero days.

Supplementary Method 1: Evaluation of eDNA deposition

Generally, eDNA is deposited (and re-suspended) in rivers with a positive correlation between discharge and the maximum transport distance prior to deposition[2]. In the medium-sized Zeller Ache with its 0.2 to 2.5 m^3/s discharge during the study period, about 50% of the emitted eDNA should theoretically remain in the water column 500 - 1,000 m downstream of the source[2,3]. However, the riverbed in the Zeller Ache as shown in Supplementary Information 1 consisted of coarse substrate (sections I, III, VII), large boulders (sections IV and VI), or was paved (section II), all of which are non-favourable to eDNA deposition[4]. In this environment, it was not possible to pre-estimate eDNA deposition rates based on previous studies. Therefore, eDNA deposition rates were included via arbitrarily selected distance penalties of 1%, 5%, and 10% per 100 m distance between the counted fish and point 1 in initial data analysis. For example, if 1% of the emitted eDNA is supposedly deposited within 100 m of downstream transport, fish counts obtained 200 m upstream of point 1 were multiplied by 0.98.

The resulting data and respective model fits did not differ significantly between the 1%, 5%, and 10% distance penalty ($P > 0.05$ for all comparisons[5,6]), which made the inclusion of such a low, arbitrarily selected distance penalty into the modelling process hard to justify. Consequently, any deposition effects were omitted from the model ranking process.

Supplementary Method 2: Filter processing, DNA extraction, amplification, visualisation, and quantification

All laboratory work was carried out in a clean-room laboratory at the University of Innsbruck including separate rooms for DNA extraction, PCR preparation, and post-PCR analysis.

Lysis

First, lysis of samples was done through pipetting a mixture of 190 µl TES buffer (0.1 M TRIS, 10mM EDTA, 2% sodium dodecyl sulphate; pH 8) and 10 µl Proteinase K (VWR, 20 mg/ml) on each filter followed by vortexing and incubation overnight on a rocking platform at 56 °C. The day after, filters were transferred to a plastic insert positioned in a 2 ml reaction tube using DNA-free plastic sticks. Lysates (400-900 µl of lysis buffer and rest water contained in the filter) were centrifuged (10 min, 14 000 rpm) through the perforated bottom of the insert into the reaction tube.

DNA extraction

DNA extraction was carried out with the Biosprint 96 robotic platform (QIAGEN) using an optimised DNA uptake protocol: lysates were split up into three binding plates (96-well) to extract DNA from a maximum of 900 µl. Wells for sample processing in each binding plate contained 300 µl AL buffer (QIAGEN), 300 µl Isopropanol and a maximum of 300 µl lysate. Wells that contained less than 300µl of lysate were filled up with TES buffer. Additionally, 30 µl MagAttract magnetic beads (QIAGEN) were added to the first binding plate. Four negative controls, which contained 300 µl AL buffer, 300 µl Isopropanol and 300 µl TES buffer, were incorporated into every binding plate. Via consecutive uptake steps on the robotic platform, DNA was combined into one binding plate. In a second step, this plate was used for extraction with the Biosprint 96 blood & tissue protocol (QIAGEN) in accordance with the manufacturer's instructions, except for elution into 100 µl 1 × TE-buffer instead of AE-buffer.

PCR, amplicon visualisation, and absolute quantification

DNA of *A. mento* and *V. vimba* was amplified via species-specific primers (Supplementary Method 2 Tab. 1) in singleplex endpoint PCRs coupled with capillary electrophoresis (CE-PCRs) and digital PCRs. The Multiplex PCR Kit (QIAGEN) was used for endpoint PCRs and each 10 µl reaction contained 1 × reaction mix, 0.5 µM of forward and reverse primer, 5 µg BSA, 30 mM TMAC and 3.2 µl of DNA extract. CE-PCR conditions were: denaturation at 95 °C for 15 min, followed by 35 cycles of 94 °C for 30 s, 66 °C for 3 min and 72 °C for 60 s, and final elongation at 72 °C for 10 min. Each PCR plate contained two negative controls (PCR-grade water instead of DNA extract), and two positive controls (DNA extracts of the target species)

and 92 field samples, which were screened in random order. Endpoint PCR products were visualised via the capillary electrophoresis system QIAxcel Advanced, the corresponding software QIAxcel ScreenGel (version 1.4.0, QIAGEN) and method AM320 with 30 s injection time. PCR products of the expected fragment length with a signal strength ≥ 0.08 relative fluorescence units (RFU) were deemed positive and their exact RFUs recorded subsequently. Whilst all extraction and PCR negative controls resulted negative, filtering negative controls resulted in RFUs ≥ 0.08 on six days. To control for sample cross-contamination via filtering equipment, the RFUs of *A. mento* and *V. vimba* obtained from the affected samples were separately down-corrected by the respective values of the filtering negative controls (RFUs of affected field samples: >2.1; RFUs of positive filtration controls: <0.23).

Digital PCRs contained 1 × ddPCR EvaGreen Supermix (Bio-Rad), 250 nM of *V. vimba* forward and reverse primer or 150 nM of *A. mento* forward and reverse primer and 1.1 µl (field sample) or 7 µl (MilliQ filtration control) of DNA extract. Additionally, 5% DMSO was included in *A. mento* digital PCRs and PCR-grade water was used to obtain the total volume of 20 µl used for droplet generation on the AutoDG (Bio-Rad) with Droplet Generation Oil for EvaGreen (Bio-Rad). Digital PCR conditions were: denaturation at 95 °C for 5 min, followed by 40 cycles (*V. vimba*) or 45 cycles (*A. mento*) of 95 °C for 30 s, 62 °C (*V. vimba*) or 60 °C (*A. mento*) for 1 min and 72 °C for 1 min, and final stabilisation at 4 °C for 5 min followed by 90 °C for 5 min. Digital PCR conditions (reaction mix and cycling protocol) were optimised for minimum "rain" and maximum fluorescence differences between target DNA and background signal. One negative control (PCR-grade water instead of DNA extract), and one positive control (DNA extracts of the target species) was included in each plate and samples were screened in random order. Target fluorescence was measured on the QX200 Droplet Reader (Bio-Rad) in combination with the Quanta Soft Analysis Pro Software 1.0.596 (Bio-Rad). Specificity of the digital PCR assays was confirmed with DNA extracts (~5,000 target copies / µl) of *V. vimba*, *A. mento*, *Salmo trutta*, *Oncorhynchus mykiss*, *Squalius cephalus*, and *Rutilus meidingeri* which co-occur in the Zeller Ache[1]. All digital PCR results are based on more than 14,800 droplets per reaction and fluorescence thresholds were set manually at 8,000 and 15,000 for *A. mento* and *V. vimba* respectively, to control for differences in optimum PCR conditions and the varying effect of background fluorescence (Supplementary Method 2 Fig. 1). All extraction and PCR controls resulted negative in digital PCR; to control for contamination in filtering negative controls (1.2 – 1.31 copies / µl extract occurring on the same days as previously found with CE-PCR), concentrations of *A. mento* and *V. vimba* were separately down-corrected for the affected samples.

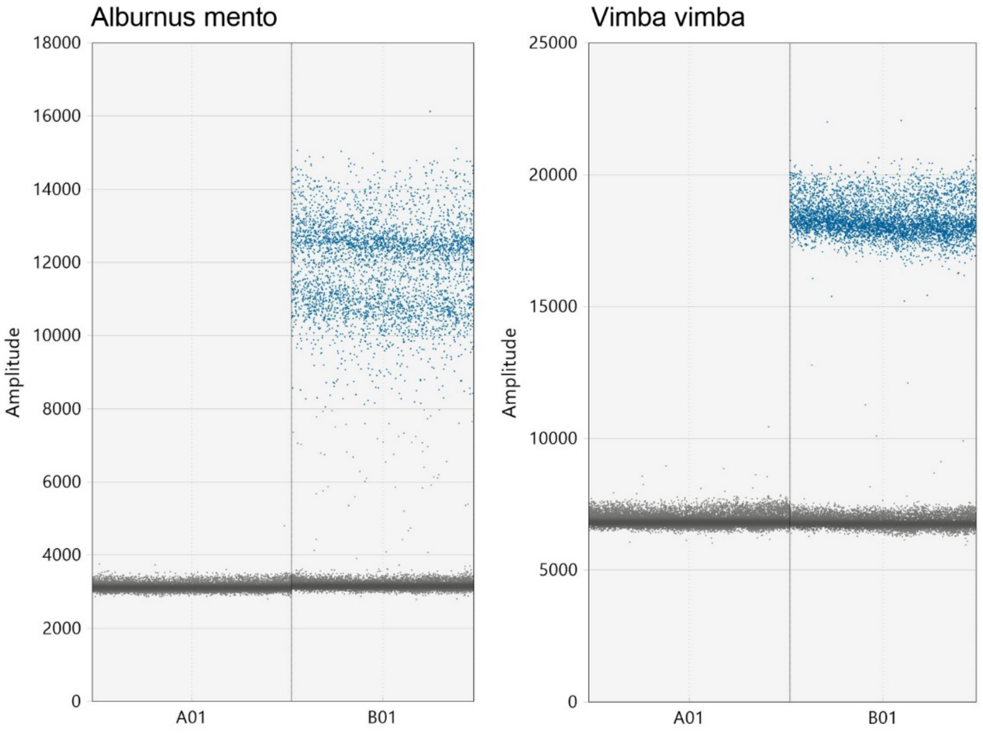

Supplementary Method 2 Figure 1: Digital PCR results obtained from negative (A01) and positive (B01) field samples tested for *Alburnus mento* (left panel) and *Vimba vimba* (right panel). Droplets containing target DNA are blue, droplets not containing target DNA are grey. The general fluorescence amplitudes of positive and negative droplets are not uniform between target species due to different optimum PCR conditions.

Relative Fluorescence Units as a semi-quantitative measure of target DNA

To test whether the RFUs obtained by CE-PCR provide a suitable proxy for the amount of target DNA, dilution series of target DNA templates of known concentration (10 000 ds/µl) were prepared[7]: the whole COI region was amplified from *V. vimba* and *A. mento* tissue extracts[8], the PCR product was cleaned up, and concentration of the target DNA templates was measured in triplicate using Quant-iT PicoGreen (Invitrogen) and a VICTOR X4 Multilabel Plate Reader (PerkinElmer)[7]. Finally, the number of double stranded target fragments per µl PCR product was calculated with the DNA Calculator[7] and used as basis for dillutions. For each species, three separate dilution series were done, whereby the starting template concentration was diluted with TE buffer to 5 000, 4 500, 4 000, 3 500, 3 000, 2 500, 2 000, 1 500, 1 000, 500, and 50 ds/µl. Then, CE-PCRs were carried out as described above, but with 1 µl of diluted template DNA and 2.2 µl PCR-grade water. Per species, RFUs were correlated to template DNA double strands in PCR leading to a logarithmic relationship (Supplementary Method 2 Fig. 2).

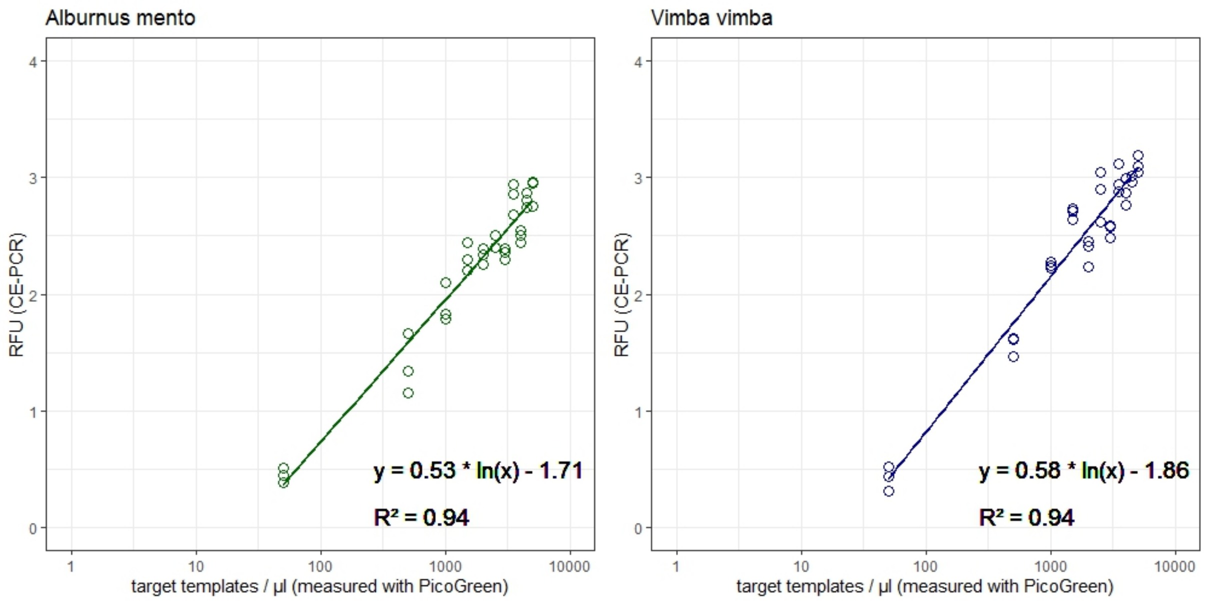

Supplementary Method 2 Figure 2: Relative fluorescence units (RFUs) obtained from three dilution series of target DNA templates with a known number of double stranded DNA of *Alburnus mento* (green) and *Vimba vimba* (blue) per µl. Each point represents the result of one CE-PCR; the number of target templates per µl was calculated using Quant-iT PicoGreen, a VICTOR X4 Multilabel Plate Reader, and the DNA Calculator[7].

In a second test, field samples obtained at point 1 were analysed with both CE-PCR and digital PCR (see "PCR, amplicon visualisation, and absolute quantification" for conditions). This test confirmed the logarithmic relationship between target copies per µl extract and RFUs. Furthermore, it showed that CE-PCR was capable of reliably detecting target DNA from a minimum of 25 – 42 ds per µl extract in filtered field samples (Supplementary Method 2 Fig. 3). The greater dispersion of individual samples from the regression line is potentially caused by the exponential nature of CE-PCR and the use of only one replicate per sample in digital PCR and CE-PCR. After these tests, RFUs were used as a proxy for target eDNA concentration in addition to absolute target DNA copies per µl extract obtained from digital PCR.

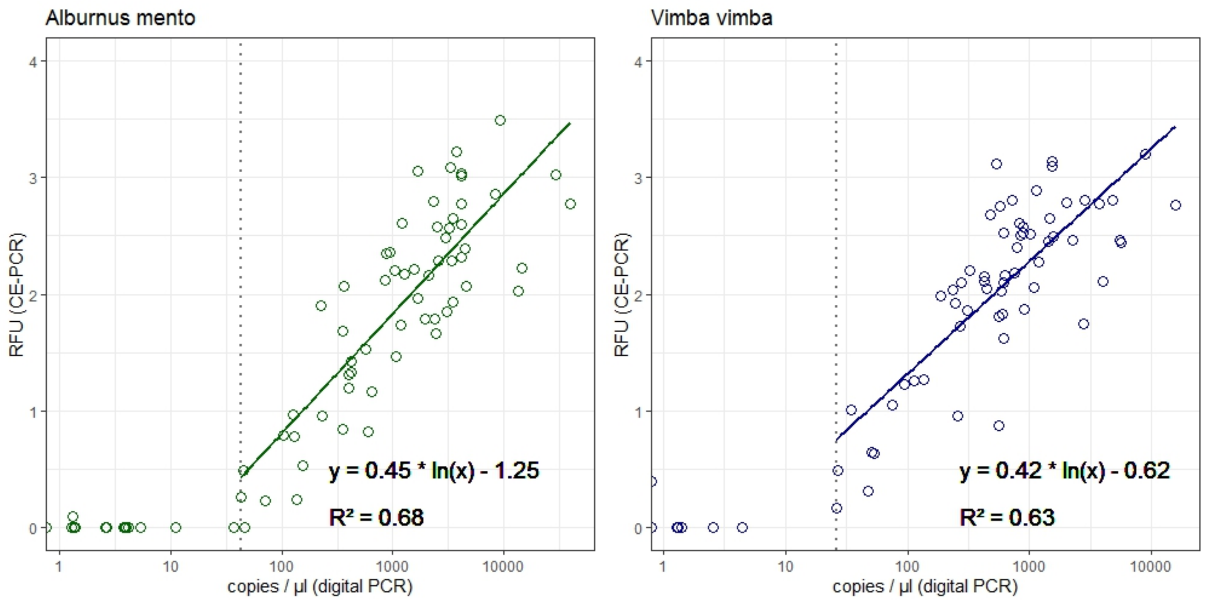

Supplementary Method 2 Figure 3: Point 1 field samples analysed via CE-PCR (RFUs) and digital PCR (copies / µl DNA extract) for *A. mento* (green) and *V. vimba* (blue). The grey dashed lines at 43 copies / µl (*A. mento*) and 26 copies / µl (*V. vimba*) represent the minimum target DNA concentration necessary for reliable detections via CE-PCR from these field samples; regression lines and equations are based on points above this detection limit.

Supplementary Method 2 Table 1: Primer combinations used for target DNA amplification. Per primer (pair), the target taxon, primer name and sequence, amplicon sizes, Tm (calculated with a modified version of Primer3 2.3.7 in Geneious Prime 2019.2.1), and the original literature source are displayed.

target taxon	primer name	primer sequence (5‘ - 3‘)	Tm (°C)	target gene	fragment length (bp)	source
Vimba vimba	Vim-vim-S676	AATCTCGCCCATGCTGGC	60.5	COI	177	Thalinger, et al. (2016)[8]
	Vim-vim-A677	GACGGCTGTTACTAGTACGGCC	62.7			
Alburnus mento	Alb-men-S662	TTTCTGACTCCTTCCGCCG	59.7	COI	200	Thalinger, et al. (2016)[8]
	Alb-men-A662	TGGTGGTAATGAAGTTGACTGCA	60.4			

Supplementary Method 3: packages and functions used for data analyses in R

The packages “VGAM”[9-11] and “AICcmodavg”[12] were used for ordinal model ranking. Tests for correlation and cross-correlation between fish count and eDNA time series were done using the package “zoo”[13], which also includes the “Ljung-Box”-test used to test for auto-correlation. The ARIMA model best describing eDNA signal strength over time was calculated using “auto-arima” (R package “forecast”[14,15]).

Supplementary References

1. Csar, D. & Gumpinger, C. *Die Migration der Fischfauna im Unterlauf von Seeache und Zeller Ache: unter besonderer Berücksichtigung der Natura 2000 Schutzgüter Perlfisch (Rutilus meidingeri) und Seelaube (Alburnus mento)* (Technisches Büro für Gewässerökologie DI Clemens Gumpinger, Wels, Austria, 2010)
2. Pont, D. *et al.* Environmental DNA reveals quantitative patterns of fish biodiversity in large rivers despite its downstream transportation. *Scientific Reports* **8**, 10361, https://doi.org/10.1038/s41598-018-28424-8 (2018).
3. Wilcox, T. M. *et al.* Understanding environmental DNA detection probabilities: a case study using a stream-dwelling char *Salvelinus fontinalis*. *Biological Conservation* **194**, 209–216, https://doi.org/10.1016/j.biocon.2015.12.023 (2016).
4. Shogren, A. J. *et al.* Controls on eDNA movement in streams: Transport, retention, and resuspension. *Scientific Reports* **7**, 5065, https://doi.org/10.1038/s41598-017-05223-1 (2017).
5. Cohen, J., Cohen, P., West, S. G. & Aiken, L. S. *Applied multiple regression/correlation analysis for the behavioral sciences*. third edn, (Lawrence Earlbaum Associates, 2003).
6. Soper, D. S. *Significance of the Difference between Two Sopes Calculator [Software]*, http://www.danielsoper.com/statcalc (2018).
7. Sint, D., Raso, L. & Traugott, M. Advances in multiplex PCR: balancing primer efficiencies and improving detection success. *Methods in Ecology and Evolution* **3**, 898–905, https://doi.org/10.1111/j.2041-210X.2012.00215.x (2012).
8. Thalinger, B. *et al.* Molecular prey identification in Central European piscivores. *Molecular Ecology Resources* **16**, 123–137, https://doi.org/10.1111/1755-0998.12436 (2016).
9. Yee, T., W. *Vector generalized linear and additive models: With an implementation in R.*, (Springer, 2015).
10. Yee, T., W. VGAM: Vector generalized linear and additive models. R package version 1.0-5., https://CRAN.R-project.org/package=VGAM (2018).
11. Yee, T., W. & Wild, C. J. Vector generalized additive models. *Journal of Royal Statistical Society, Series B* **58**, 481–493 (1996).
12. Mazerolle, M. J. AICcmodavg: Model selection and multimodel inference based on (Q)AIC(c). *R package version 2.2-2,* https://cran.r-project.org/package=AICcmodavg (2019).
13. Zeileis, A. & Grothendieck, G. zoo: S3 infrastructure for regular and irregular time series. *Journal of Statistical Software* **14**, 1–27, doi: 10.18637/jss.v014.i06 (2005).
14. Hyndman, R. *et al.* Forecast: forecasting functions for time series and linear models. *R package version 8.3*, http://pkg.robjhyndman.com/forecast (2018).
15. Hyndman, R. & Khandakar, Y. Automatic time series forecasting: the forecast package for R. *Journal of Statistical Software* **26**, 1–22, http://www.jstatsoft.org/article/view/v027i03 (2008).
